# Nrf2 and STAT3 Alleviates Ferroptosis-Mediated IIR-ALI by Regulating SLC7A11

**DOI:** 10.1155/2020/5146982

**Published:** 2020-09-18

**Authors:** Zhuanzhuan Qiang, Hui Dong, Yangyang Xia, Dongdong Chai, Rong Hu, Hong Jiang

**Affiliations:** Shanghai Ninth People's Hospital, Shanghai Jiao Tong University School of Medicine, Center for Specialty Strategy Research of Shanghai Jiao Tong University China Hospital Development Institute, China

## Abstract

Acute lung injury (ALI) has gained increased attention in the field of critical illness research and is associated with a fatality rate of approximately 50%. Nuclear factor erythroid 2-related factor2 (*Nrf2*) is a key regulator of intracellular oxidation homeostasis and also functions as an antioxidant. It has been reported that Nrf2 associated antioxidant stress is closely related to ferroptosis inhibition. Signal transducer and activator of transcription 3 (STAT3) is activated into phosphorylated STAT3 (pSTAT3) in response to tissue damage and serves as a warning signal to enhance the inflammatory response. In this study, an intestinal ischemia/reperfusion-induced acute lung injury (IIR-ALI) model was established in C57BL/6 mice to investigate the role of Nrf2 in regulating IIR-ALI-associated ferroptosis. Compared with those in the IIR-ALI group, the injection of Fe (15 mg/kg) or ferrostatin-1 (5 mg/kg) (ferroptosis promoter and inhibitor, respectively) via the tail vein could aggravate or alleviate lung injury and pulmonary edema, respectively. Nrf2 was increased in IIR-ALI and promoted the phosphorylation of STAT3 to amplify downstream signals. An *in vitro* oxygen-glucose deprivation and reoxygenation (OGD-R) model was established in MLE12 cells to imitate the ischemia/reperfusion condition. The cells were transfected with lentiviruses to increase or downregulate the levels of STAT3. We found that Nrf2 and STAT3 played key roles in ferroptosis by regulating SLC7A11, which improved the pathological processes associated with ALI.

## 1. Introduction

Intestinal ischemia reperfusion (IIR) injury is associated with a high mortality rate and occurs in numerous clinical pathologies, including small intestinal volvulus, acute mesenteric ischemia, shock, and trauma. [1, 2]. Massive epithelial cell death is a major cause of intestinal mucosal barrier dysfunction, which leads to systemic inflammation and the dysfunction of remote organs [3]. Moreover, severe intestinal damage caused by IIR may lead to acute injury of remote organs (e.g., lung and liver), which plays a critical role in prognosis [4]. Recently, IIR injury events have increasingly been shown to involve nonapoptotic pathways (e.g., necroptosis [5, 6], pyroptosis [7, 8], and ferroptosis [9, 10]). Thus, the physiopathological mechanism of acute lung injury (ALI) caused by IIR attracted our attention.

Ferroptosis is a recently recognized form of regulated cell death (RCD), which is characterized as iron-dependent and caspase-independent nonapoptotic cell death [11]. Ferroptosis differs from other classical nonapoptotic cell death programs by its characteristics of mitochondrial shrinkage and increased mitochondrial membrane density, iron and lipid reactive oxygen species (L-ROS) accumulation, and the involvement of a unique set of genes [12]. The biochemical mechanism underlying ferroptosis is the iron-catalyzed formation of lipid radicals combined with glutathione (GSH) depletion or the inactivation of the lipid repair enzyme GSH peroxidase 4 (GPx4) [13, 14]. Ferroptosis can be prevented by lipophilic antioxidants (e.g., CoQ10, vitamin E, ferrostatins, and liproxstatins) [15]. The small-molecule, ferrostatin-1 has been identified as a specific inhibitor of ferroptosis [11]. Moreover, ferroptosis has been implicated in several pathophysiological contexts, including degenerative diseases, tumor suppression, antiviral immunity, and stroke [16]. Recent studies have indicated that ferroptosis is closely associated with IIR injury, which can exaggerate IIR-induced lung and liver injury [4]. However, the intrinsic mechanism remains unexplored. In this study, we sought to explore the role of ferroptosis in the pathogenesis of acute lung injury (ALI).

Nuclear factor E2-related factor 2 (*Nrf2*) is the key regulatory factor required for cells to maintain an oxidative steady state and is activated under conditions of high oxidative stress [17]. Nrf2 has several target genes, including intracellular redox-balancing proteins like heme oxygenase-1 (HO-1), glutathione peroxidases (GPX), and SLC7A11 [18, 19]. These downstream factors play crucial roles in cellular defense mechanisms. It has been reported that Nrf2 protects against cerebral [20], liver [21], cardiac [22], and intestinal IR damage [23, 24]. Moreover, Nrf2 inhibition reverses ferroptosis resistance associated with antitumor drug treatment [25–27]. However, the detail role of Nrf2 in IR-induced ferroptosis remains unclear.

STAT3 is a member of the STAT family that plays a critical role in both inflammation and tumorigenesis [28]. STAT3 activation (phosphorylated STAT3 (pSTAT3)) increases the permeability of the lysosomal membrane and promotes cell survival in erastin-induced ferroptosis in breast cancer cells [29]. In addition, the SLC7A11 gene belongs to the solute transport family and encodes a cystine/glutamate xCT transporter, which is a key gene involved in regulating “iron overload-ferroptosis” [12]. Linher et al. found that STAT3 activation is coupled with increased system X_c_^−^ activity in human breast cancer cells and proposed that targeting system X_c_^−^ together with STAT3/5 inhibitors may improve therapeutic anticancer effects [30, 31].

In this study, we hypothesized that ferroptosis participated in IIR-induced ALI (IIR-ALI) and that the inhibition of Nrf2 during ischemia contributed to ferroptosis following reperfusion through STAT3 signaling. We sought to elucidate whether STAT3 can alleviate ferroptosis in IIR-ALI through regulating SLC7A11. This study is aimed at providing novel insight and targets for the treatment of IIR-ALI.

## 2. Materials and Methods

### 2.1. Animals

Eight-week-old C57BL/6J mice (purchased from the Animal Center of Shanghai Jiao Tong University School of medical, Shanghai, China) and 8-week-old Nrf2 knockout (*Nrf2^−/−^*) mice with the same genetic background (provided by the RIKEN Bio-Resource Centre through the National Bio-Resource Project, MEXT, Japan) were used to conduct *in vivo* experiments. Mice were housed under controlled temperature (21°C ± 2°C) and humidity (60% ± 5%) on a 12 h light/dark cycle. The mice were fed standard mouse chow and water ad libitum. All experiments were conducted in accordance with the NIH guidelines and approved by the Ethical Committee of Shanghai Ninth People's Hospital for Animal Research.

### 2.2. IIR Mouse Model

The mice were randomly assigned to four groups: (*n* = 6/group): (1) sham, (2) IIR, (3) IIR+Fe, and (4) IIR+Fer-1. All mice were anesthetized by an intraperitoneal (i.p.) injection of pentobarbital (50 mg/kg body weight) and permitted to breathe spontaneously during surgery. The superior mesenteric artery was exposed in the sham group without occlusion. To establish the IR model, a midline laparotomy was performed and the superior mesenteric artery was occluded with a microvascular clamp for 45 min and perfusion was recovered for 180 min. Prior to blocking the superior mesenteric artery, 15 mg/kg Fe-citrate (III) (Fe) (CAS 2238-05-8, Sigma-Aldrich, USA) and 1.5 mg/kg ferrostatin-1 (Fer-1) (CAS 347174-05-4, Sigma-Aldrich, USA) were injected into the tail vein of the IIR+Fe and IIR+Fer-1 groups, respectively. All mice were resuscitated with an intraperitoneal injection of normal saline (1.0 mL) after surgery. Animals were sacrificed following 3 h of reperfusion, and the tissues were harvested. Tissues were snap frozen in liquid nitrogen and stored at -80°C until further experimental analysis.

### 2.3. Lung Wet/Dry Weight Ratio

Fresh lung samples were dissected and weighed immediately to obtain the wet weight. The lungs were placed in a drying oven at 65°C for 72 h until a constant weight was obtained. The wet to dry weight ratio was calculated to represent the degree of lung edema [32].

### 2.4. Histology and Scoring of Lung Injury

The lung samples were lavaged with normal saline, dissected, and fixed in a 4% paraformaldehyde solution at room temperature for 24 h. Lung tissues were embedded in paraffin, cut into 5 *μ*m sections, and stained with hematoxylin and eosin (Solarbio, Beijing, China). The evaluation of lung injury was performed in a blinded fashion according to the modified scoring system described by Jain et al. [33]. The scoring system was comprised of four independent parameters: (1) vascular congestion, (2) hemorrhage, (3) aggregation of cellular infiltration, and (4) thickening of the alveolar wall (0—normal lung; 1—lesions involving less than 25% of the lung, with slight injury; 2—lesions involving 25%–50% of the lung, with moderate injury; 3—lesions involving 50%–75% of the lung, with severe injury; and 4—lesions involving 75% or more of the lung, with extremely severe injury). The sum of the scores for the four parameters was calculated.

### 2.5. Transmission Electron Microscopy

The lung tissues were fixed for 2 h in 2.5% glutaraldehyde in a 0.05 M sodium cacodylate buffer at a pH of 7.2 at 25°C, followed by 2 h in 2% OsO_4_ in a 0.1 M sodium cacodylate buffer and 18 h in 1% aqueous uranyl acetate. After dehydration through an ethanol series, the specimens embedded in Epon 812 and ultrathin sections were collected on copper grids. After staining with uranyl acetate and lead citrate, the sections were examined using a Tecnai G2 spirit BioTwin transmission electron microscope (FEI Company, Hillsboro, Oregon).

### 2.6. Cell Culture, Oxygen-Glucose Deprivation (OGD), and Reoxygenation Model Procedures

MLE12 cells were purchased from the cell bank of the Chinese Academy of Sciences (Shanghai, China) and cultured in a humidified incubator maintained at 37°C and 5% CO_2_ in Dulbecco's modified Eagle's medium (11965, Gibco, USA) containing 10% fetal bovine serum (0500, Gibco, USA), penicillin (100 IU/mL), and streptomycin sulphate (100 *μ*g/mL). To establish a model of hypoxia, the cells were incubated in a microaerophilic system (Thermo, WA, USA) with 5% CO_2_ and 95% N_2_ gas. For reoxygenation, the cells were cultured under normoxic conditions.

### 2.7. Cell Viability Assay

Cell viability was evaluated using a Cell Counting Kit-8 (CCK-8, CK04, Dojindo, Tokyo, Japan) assay according to the manufacturer's instructions. The cells were plated in 96-well plates at a density of 5000 cells/well. After plating (24 h), the cells were subjected to various treatments for the indicated time points. The CCK-8 solution (10 *μ*L) was added to each well, and the cells were incubated for another 3 h at 37°C. The optical density (OD) values were measured at 450 nm using a microplate reader. The cells that stained positive with the CCK-8 solution were considered viable and are presented as a percentage compared with the control cells. Each assay was performed in triplicate.

### 2.8. Western Blotting

Lung tissues and cells were homogenized and incubated in lysis buffer containing a protease inhibitor cocktail. The protein concentration was measured using a BCA kit (KGA902, KeyGEN BioTECH, Nanjing, China), and the proteins were then denatured at 100°C for 5 min. The proteins were loaded onto a 10% SDS-PAGE gel and transferred to a polyvinylidene fluoride (PVDF) membrane. The blots were blocked with 5% nonfat milk at room temperature for 1 h and incubated with primary antibodies overnight at 4°C. The primary antibodies included rabbit monoclonal anti-Nrf2 (ab137550, Abcam, 1 : 1,000), anti-SLC7A11 (ab37185, Abcam, 1 : 1,000), anti-STAT3 (ab68153, Abcam, 1 : 2,000), anti-pSTAT3 (ab76315, Abcam, 1 : 2,000), and anti-*β*-actin (4970S, Cell Signaling Tech, 1 : 1,000). After washing three times with TBST for 15 min, the strips were incubated with anti-mouse or anti-rabbit horseradish peroxidase- (HRP-) conjugated secondary antibodies and detected using an ECL detector. The signals were scanned and quantified using ImageJ software.

### 2.9. Transient Transfection

M_STAT3-shRNA (PGMLV-SC5), Scramble-SC5 Lentivirus, PGMLV-CMV-M_STAT3-3×Flag-PGK-Puro, and Puro lentivirus vectors were purchased from Genomeditech (Shanghai, China). The lentiviral vectors were transfected into MLE12 cells, and both the Puro lentivirus and Scramble-SB3 Lentivirus were used as negative controls. For the stable silencing and overexpression of the STAT3 gene, the cells were plated into six-well plates at a density of 2.5 × 10^5^ cells/well and allowed to adhere and grow to a confluence of approximately 40%-50% overnight. The following day, the cells were transfected with lentiviruses using the protocol provided by the manufacturer. Stably transfected cells were selected using 2 *μ*g/mL puromycin (1299MG025, BioFroxx, Germany). The silencing efficiency was confirmed by a Western blot and qRT-PCR following transfection for 72 h.

### 2.10. MDA Assay

MDA was evaluated using an MDA assay kit (MAK085, Sigma-Aldrich, USA) according to the manufacturer's instructions. Tissue (10 mg) or cells (1 × 10^6^) were homogenized on ice in 300 *μ*L MDA Lysis Buffer containing 3 *μ*L BHT (100×). The samples were centrifuged at 13,000 × *g* for 10 min to remove any insoluble material. Then, 600 *μ*L of the TBA solution was added to each vial containing samples. The samples were incubated at 95°C for 60 min and cooled to room temperature in an ice bath for 10 min. Then, 200 *μ*L of each reaction mixture was pipetted into a 96-well plate and the absorbance was measured at 532 nm (A _532_).

### 2.11. GSH Assays

GSH was measured using a total GSH assay kit (BC1175, Solarbio, China) according to the manufacturer's protocol. The lung tissue or 1 × 10^8^ cells were washed with PBS then centrifuged at 600 × *g* to obtain a compacted cell pellet. The supernatant was removed, and the volume of the pellet was measured. Then, 3 volumes of 5% SSA Solution were added to the cell pellet and vortexed. The suspension was freeze-thawed twice (liquid nitrogen was used to freeze and a 37°C bath to thaw) and left for 5 min at 2°C-8°C. The extract was centrifuged at 10,000 × *g* for 10 min, and 200 *μ*L was added to a 96-well plate to measure the absorbance at 412 nm (A _412_).

### 2.12. Iron Assays

Determination of the endogenous total, ferric, and ferrous iron levels of the lung tissue in the IIR-ALI model was carried out using the iron assay kit (ab83366; Abcam) according to the manufacturer's instructions. Lung tissue was washed in cold PBS and homogenized in iron assay buffer on ice using a Dounce homogenizer, centrifuged at 16000 × *g* for 10 min, and the supernatant was collected for the iron assay. 25 *μ*L of samples was made up to 100 *μ*L in a 96-well plate with assay buffer and incubated for 30 min at 37°C with a 5 *μ*L iron reducer (for total iron) or assay buffer (for ferrous iron) along with standards. 100 *μ*L of iron probe was added to each reaction, mixed, and incubated for a further 60 minutes at 37°C in the dark. The absorbance at 593 nm, which corresponds to the iron levels, was determined using a microplate reader.

### 2.13. Statistical Analysis

Data were expressed as the mean ± SEM. Differences were analyzed using an unpaired two-sided Student's *t*-test for a two-group comparison or one-way ANOVA test followed by a Bonferroni post hoc test for multiple comparisons. A two-way ANOVA was conducted to examine the effects of two independent variables. Statistical significance was set at *P* < 0.05. SPSS statistical software 20.0 for Windows was used for the analysis.

## 3. Results

### 3.1. Ferroptosis Occurred in ALI due to IIR

**Figure 1:**
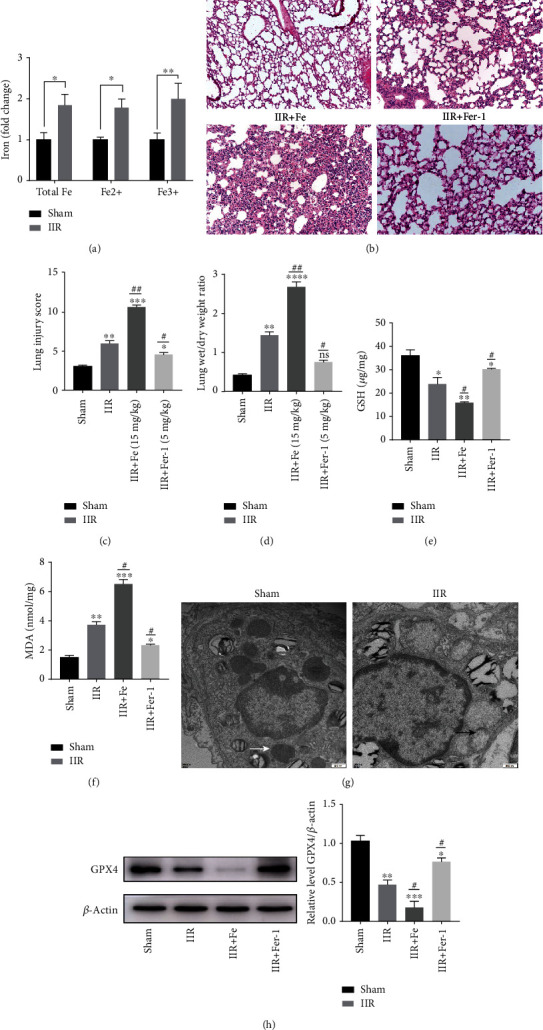
Ferroptosis occurred in ALI due to IIR. (a) Endogenous levels of Fe of the lung tissue in the IIR-ALI model was measured with an iron assay. ^∗^*P* < .05, ^∗∗^*P* < .01, and ^∗∗∗^*P* < .001 between the groups. (b) Mice were divided into four groups: sham, IIR, IIR+Fe, and IIR+Fer-1. HE staining of the lung tissues (*n* = 6). (c, d) The lung pathological damage score following the administration of Fe (15 mg/kg) and Fer-1 (5 mg/kg), respectively (*n* = 6 per group). (e) GSH level in each group was measured by GSH assay (*n* = 6 per group). (f) MDA level in each group was measured by MDA assay (*n* = 6 per group). (g) Representative TEM images. The white arrow indicates the ultrastructure of the mitochondria in the sham group. The black arrows indicate the reduction or disappearance of mitochondrial cristae in the IIR group (*n* = 3 per group). (h) The level of lipid peroxidation-associated index, GPX4, in each group and the representative quantification of GPX4 protein. ^∗^*P* < .05, ^∗∗^*P* < .01, ^∗∗∗^*P* < .001, ^#^*P* < .05, ^##^*P* < .01, and ^###^*P* < .001 between the groups. ^∗^Compared with the sham group. ^#^Compared with the IIR group.

**Figure 2:**
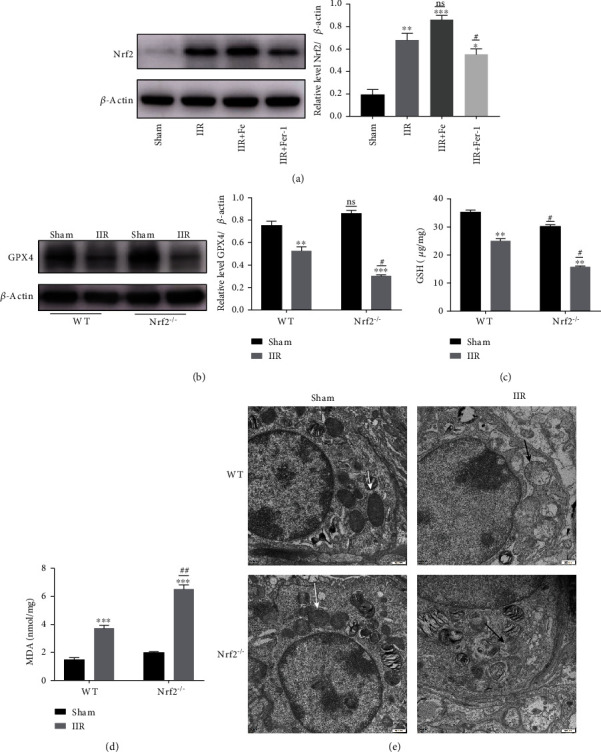
The lipid peroxidation index associated with ferroptosis was significantly altered in IIR-induced ALI. The mice were divided into four groups: sham, IIR, IIR+Fe, and IIR+Fer-1 after administrating Fe (15 mg/kg) and Fer-1 (5 mg/kg), respectively. (a) The level Nrf2 protein expression in each group was measured with a western blot and the representative quantification of Nrf2 protein (*n* = 6 per group). (b) The level of GPX4 in WT and *Nrf2^−/−^* group and the representative quantification of GPX4 protein (*n* = 6 per group). (c) The GSH level in the WT and *Nrf2^−/−^* group (*n* = 6 per group). (d) The MDA level in WT and *Nrf2^−/−^* group (*n* = 6 per group). (e) Representative TEM images of WT and *Nrf2^−/−^* mice. The white arrow indicates the ultrastructure of the mitochondria in the sham group, and the black arrows indicate the reduction or disappearance of mitochondrial cristae in the IIR group (*n* = 3 per group). ^∗^*P* < .05, ^∗∗^*P* < .01, ^∗∗∗^*P* < .001, ^#^*P* < .05, and ^##^*P* < .01 between the groups. ^∗^Compared with the sham group. ^#^Compared with the IIR group.

To verify the existence of ferroptosis and its role in IIR-ALI, we determined whether the inhibition of ferroptosis could rescue IIR-ALI. Firstly, the endogenous level of ferric and ferrous iron in lung tissue was tested in the IIR-ALI model. We found that total Fe, Fe^2+^, and Fe^3+^ in IIR group were significantly increased compared with the sham group (Figure 1(a)). Ferrostatin-1, a specific ferroptosis inhibitor that has previously been shown to attenuate IR damage [9, 34], was administered to mice to determine the role of ferroptosis inhibition on IIR injury. *In vivo* ferroptosis inhibition by ferrostatin-1 substantially attenuated pulmonary epithelial cell damage and lung permeability as evidenced by H&E staining and W/D experiments (Figures 1(b) and 2(d)). The wet to dry ratio of the lung tissue exhibited an increase following IIR, whereas the administration of Fe (15 mg/kg) and Fer-1 (5 mg/kg) could, respectively, increase and decrease the ratio accordingly (Figure 1(d)). Some core factors (e.g., MDA and GSH) have been accepted as a pivotal and valid metabolic index for the regulation of ferroptosis [14, 35]. GSH, an important intracellular antioxidant factor, was reduced in the IIR model, and the reduction was more obvious with the addition of Fe; however, the reduction was alleviated following the addition of Fer-1 (Figure 1(e)). Moreover, the lipid peroxidation product, MDA, increased in the IIR model, which was more obvious after adding Fe, and reduced after adding Fer-1 (Figure 1(f)). The reduction or disappearance of mitochondrial cristae is a characteristic indicator of ferroptosis [4, 12]. Thus, we next used TEM (transmission electron microscopy) to investigate the morphological features of ferroptosis. We observed that compared with the sham group, the mitochondrial morphology in the alveolar epithelial cells of mice in the IIR group underwent the characteristic changes of ferroptosis, including the presence of smaller mitochondria and cristae reduction (Figure 1(g)). Furthermore, the expression of GPX4 (a key ferroptosis factor [14] related to lipid peroxidation) was significantly reduced in the IIR model. This reduction was more significant in the Fe group, which was recovered in the Fer-1 group (Figure 1(h)). Therefore, these findings suggest that ferroptosis occurred in IIR-ALI.

### 3.2. Nrf2 Upregulation Inhibits Ferroptosis and Plays a Protective Role in IIR-ALI

Nrf2 is an important regulator of antioxidative stress; we found that the expression of Nrf2 was significantly increased in the IIR model. This increase was more severe in the Fe group, which recovered after an administration of Fer-1 (Figure 2(a)). To further explore the role of *Nrf2* in ferroptosis and IIR-ALI, we used *Nrf2* gene knockout (*Nrf2^−/−^*) mice. We found that the expression of GPX4 was significantly reduced in the IIR model. This reduction was more significant in the *Nrf2^−/−^* group (Figure 2(b)). Furthermore, we detected the level of GSH and MDA. The results show that GSH was reduced in the IIR model, and the reduction was more obvious in the *Nrf2^−/−^* group (Figure 2(c)) and the lipid peroxidation product, MDA, increased much more serious in the *Nrf2^−/−^* group after IIR (Figure 2(d)). The mitochondria observed via TEM had decreased in size, the cristae had decreased or disappeared, and the outer membrane had ruptured in the *Nrf2^−/−^* IIR group, which suggested that Nrf2 could alleviate ferroptosis (Figure 2(e)). This finding is also consistent with that of previous studies showing that Nrf2 plays a protective role in ALI [32]. Interestingly, we found that Nrf2 might alleviate ALI through inhibiting ferroptosis.

### 3.3. Nrf2 Regulates Activation of STAT3 in IIR-ALI Ferroptosis

**Figure 3:**
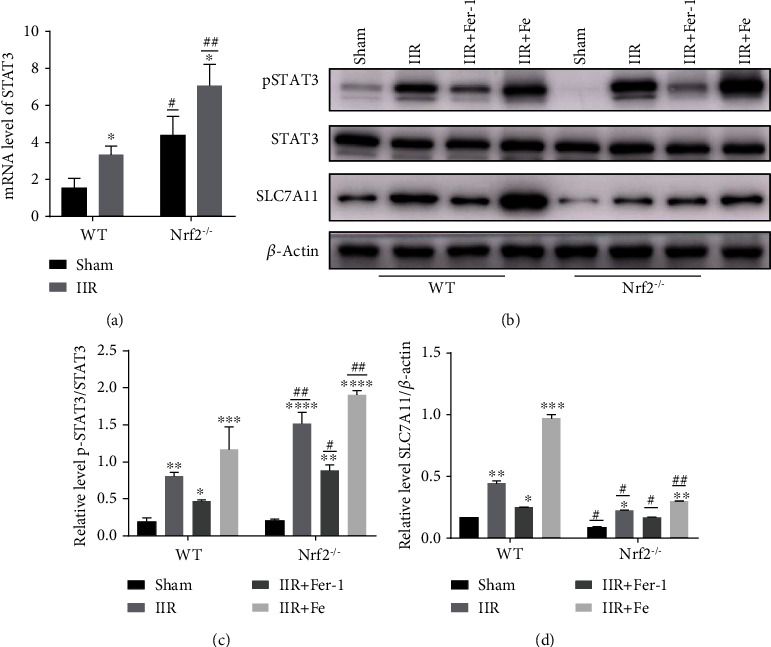
pSTAT3 and the downstream protein, SLC7A11, were significantly increased in IIR-ALI and regulated by Nrf2. (a) The level of STAT3 mRNA in the *Nrf2^−/−^* mice compared with WT. ^∗^*P* < .05, ^∗∗^*P* < .01, ^∗∗∗^*P* < .001, ^#^*P* < .05, and ^##^*P* < .01 between the groups. ^∗^Compared with the sham group; ^#^*Nrf2^−/−^*compared with that of the WT group (*n* = 6 per group). (b) WT and *Nrf2^−/−^* mice were divided into four groups: sham, IIR, IIR+Fe, and IIR+Fer-1. The level of STAT3, pSTAT3, and SLC7A11protein expression was measured using a western blot. (c) The representative quantification of pSTAT3 protein, normalized to the corresponding level of total STAT3 (*n* = 6 per group). (d) The representative quantification of SLC7A11 protein, normalized to the corresponding level of *β*-actin (*n* = 6 per group). ^∗^*P* < .05, ^∗∗^*P* < .01, ^#^*P* < .05, and ^##^*P* < .01 between the groups. ^∗^Compared with the sham group; ^#^*Nrf2^−/−^*compared with that of the WT group.

To further explore whether Nrf2 regulates STAT3 in ferroptosis-mediated IIR-ALI, *Nrf2* gene knockout mice were used. RT-PCR and a western blot were performed on the lung tissues of both the *Nrf2^−/−^* and WT mice in each of the four groups, respectively. The RT-PCR results showed that the mRNA level of STAT3 was significantly increased in the *Nrf2^−/−^* mice compared with the WT mice (Figure 3(a)). Although the protein level of total STAT3 was unchanged, STAT3 phosphorylation was significantly increased in the *Nrf2^−/−^* mice compared with the WT mice in four groups, indicating activation of the STAT3 antioxidant pathway (Figures 3(b) and 3(c)). Similarly, the expression of the above three proteins could be increased and reduced following the administration of Fe and Fer-1, respectively (Figure 3(b)). Additionally, SLC7A11 was downregulated after knockout of *Nrf2* (Figures 3(b) and 3(d)).

### 3.4. Ferroptosis Is Present in OGD/R-Induced Lung Epithelial Cell Injury

**Figure 4:**
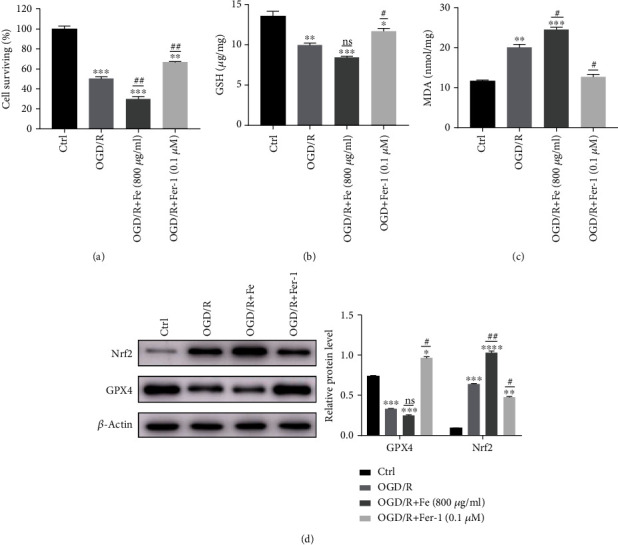
Ferroptosis occurs in OGD/R-induced lung epithelial cell damage. (a) Cell survival after oxygen-glucose deprivation (OGD) 8 h and reoxygenation (R) 12 h performed by a CCK8 assay. Cells were divided into four groups: (1) control, (2) OGD/R, (3) OGD/R+Fe (800 *μ*g/mL), and (4) OGD/R+Fer-1 (0.1 *μ*M). (b) The level of cellular GSH after OGD/R was evaluated with a GSH assay. (c) The level of cellular MDA after OGD/R was evaluated with an MDA assay. (d) The level of GPX4 and Nrf2 protein expression was measured with a Western blot. ^∗^*P* < .05, ^∗∗^*P* < .01, ^∗∗∗^*P* < .001, ^#^*P* < .05, and ^##^*P* < .01 between the groups. ^∗^Compared with the control group. ^#^Compared with the OGD/R group.

To investigate the relative contribution of ferroptosis in OGD/R-induced MLE12 cell death in vitro, we measured cell survival after OGD for 8 h and reoxygenation for 12 h in the presence or absence of various ferroptosis inhibitors or a ferroptosis promoter. Our analysis revealed that cell survival was significantly aggravated following the administration of 800 mg/mL Fe (a ferroptosis promoter) (Figure 4(a)). In contrast, administration the ferroptosis inhibitor, Fer-1, significantly reduced OGD/R-induced mortality at dose of 0.1 *μ*M (Figure 4(a)). We also evaluated the level of GSH (Figure 4(b)) and MDA (Figure 4(c)) following OGD/R. Consistent with the IIR model, GSH was reduced in the OGD/R model. While the reduction was more obvious with the addition of Fe, the reduction was alleviated with the addition of Fer-1 (Figure 4(b)). Similarly, the level of MDA increased after OGD/R, and the increase was more obvious after the addition of Fe, which could be reduced by the addition of Fer-1 (Figure 4(c)). The level of GPX4 expression was reduced after OGD/R, and the reduction was more significant in the Fe group, which was recovered in the Fer-1 group (Figure 4(d)). The expression of Nrf2 was increased in the OGD/R model and could be recovered in the Fer-1 group. These results suggest that OGD/R-induced cell death was ferroptosis related and can be inhibited by treatment with a ferroptosis inhibitor.

### 3.5. pSTAT3 Activation and Inhibitor Could Ameliorate and Aggravate OGD/R-Induced MLE12 Ferroptosis Respectably through Regulating SLC7A11

**Figure 5:**
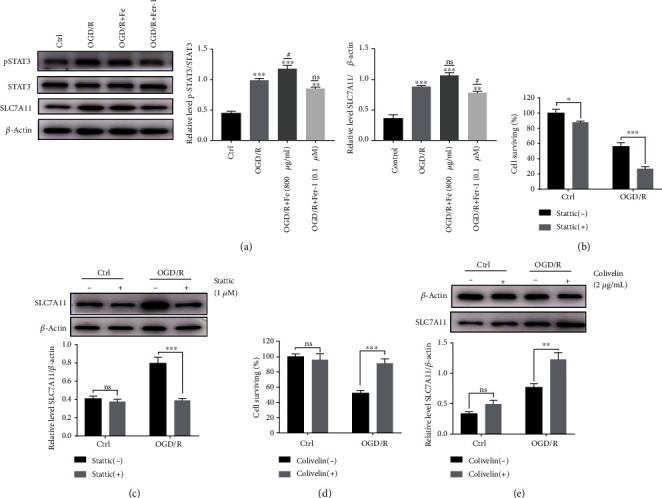
pSTAT3 upregulates SLC7A11in OGD/R-induced MLE12 ferroptosis. (a) The level of SLC7A11, STAT3, and pSTAT3 expression was detected by western blot. The intensity of pSTAT3 was normalized to the corresponding level of STAT3. The intensity of SLC7A11 was normalized to the corresponding level of *β*-actin, and the changes are shown in the histograms. ^∗^*P* < .05, ^∗∗^*P* < .01, ^∗∗∗^*P* < .001, ^#^*P* < .05, ^##^*P* < .01, and ^###^*P* < .001 between the groups. ^∗^Compared with the control group. ^#^Compared with the OGD/R group. (b) The cell proliferation activity was measured with a CCK8 assay after OGD/R and treated with pharmacologic blockade of STAT3 phosphorylation. (c) The relative level of SLC7A11 protein expression after inhibition of STAT3 phosphorylation. The SLC7A11 intensity was normalized to the corresponding level of *β*-actin, and the changes are shown in the histograms. ^∗^*P* < .05, ^∗∗^*P* < .01, and ^∗∗∗^*P* < .001 between the groups. (d) The cell proliferation activity was measured with a CCK8 assay after OGD/R and treated with pharmacologic activation of STAT3 phosphorylation. (e) The relative level of SLC7A11 protein expression after activation of STAT3 phosphorylation. The SLC7A11 intensity was normalized to the corresponding level of *β*-actin, and the changes are shown in the histograms. ^∗^*P* < .05, ^∗∗^*P* < .01, and ^∗∗∗^*P* < .001 between the groups.

Similar to the *in vivo* findings, the level of pSTAT3 and SLC7A11 protein expression in MLE12 cells increased following OGD/R and displayed increased severity following the administration of Fe (800 *μ*g/mL). On the contrary, the levels of pSTAT3 and SLC7A11 were reduced compared with that of the OGD/R group following the administration of Fer-1 (0.1 *μ*M) (Figure 5(a)). We then investigated whether activation of STAT3 (pSTAT3) could ameliorate OGD/R-induced MLE12 cell damage. Using stattic (pharmacologic inhibitor of pSTAT3) in MLE12 cell aggravate OGD/R induced MLE12 cell damage (Figure 5(b)) and downregulate SLC7A11 (Figure 5(c)). Moreover, treatment of Colivelin (pharmacologic activator of pSTAT3) could alleviate MLE12 cell damage induced by OGD/R (Figure 5(d)) and upregulate SLC7A11 (Figure 5(e)). These findings suggest that activation of STAT3 might play a protective role in ALI.

### 3.6. Nrf2-Mediated Activation of STAT3 Ameliorates OGD/R-Induced MLE12 Ferroptosis

**Figure 6:**
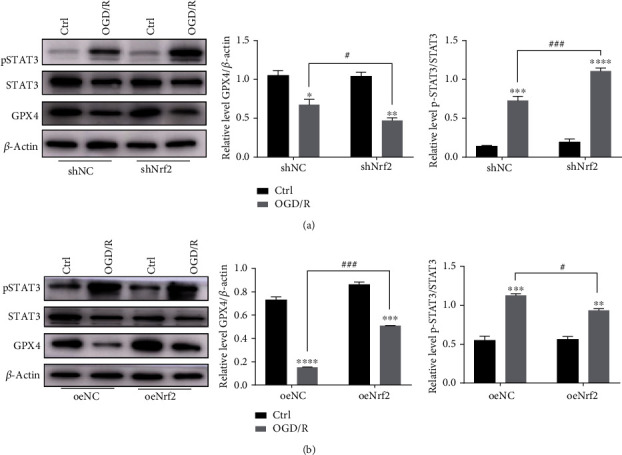
The activation of STAT3 in OGD/R-induced MLE12 ferroptosis was regulated by Nrf2. (a) Cells were transfected with Nrf2 interfering lentiviruses and then subjected to OGD/R. The level of GPX4, STAT3, and pSTAT3 expression was measured by western blot. ^∗^*P* < .05, ^∗∗^*P* < .01, ^∗∗∗^*P* < .001, ^#^*P* < .05, ^##^*P* < .01, and ^###^*P* < .001 between the groups. ^∗^Compared with the control group; ^#^shNC group compared with the shNrf2 group. (b) Cells overexpressing Nrf2 and then subjected to OGD/R. The level of GPX4, STAT3, and pSTAT3 was measured by western blot. ^∗^*P* < .05, ^∗∗^*P* < .01, ^∗∗∗^*P* < .001, ^#^*P* < .05, ^##^*P* < .01, and ^###^*P* < .001 between the groups. ^∗^Compared with the control group; ^#^shNC group compared with the shNrf2 group.

To investigate whether Nrf2 and STAT3 could inhibit ferroptosis through regulating SLC7A11 in OGD/R, lentiviruses were used to knockdown or overexpress Nrf2. The results showed that Nrf2 downregulation with a shRNA lentivirus significantly aggravated the decreased level of GPX4 and increased STAT3 phosphorylation to resist the cellular damage (Figure 6(a)). Moreover, Nrf2 overexpression alleviated the decrease in the level of GPX4 and attenuated STAT3 phosphorylation (Figure 6(b)). These data indicate that Nrf2 could regulate STAT3 activation to resist ferroptosis and plays a protective role in OGD/R-induced ferroptosis.

### 3.7. STAT3 Downregulates SLC7A11 and Aggravates OGD/R-Induced MLE12 Ferroptosis

**Figure 7:**
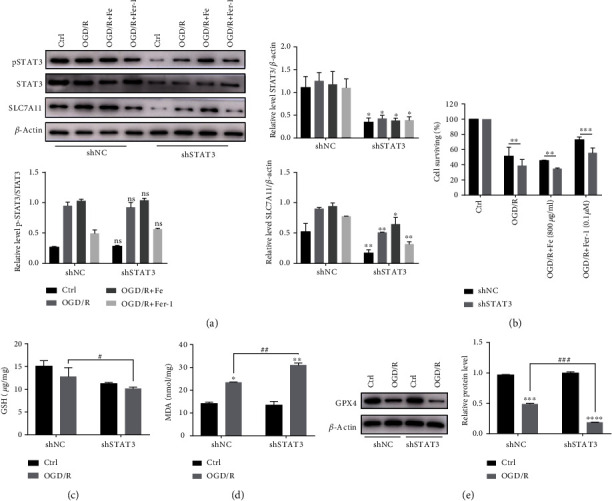
Interference with STAT3 expression may exacerbate OGD/R-induced ferroptosis. Cells transfected with the STAT3 interference lentivirus were subjected to OGD/R. (a) The relative level of STAT3, pSTAT3, and SLC7A11 protein expression after interfering with STAT3. The pSTAT3 intensity was normalized to the corresponding level of STAT3. The STAT3 and SLC7A11 intensity were normalized to the corresponding level of *β*-actin, and the changes are shown in the histograms. ^∗^*P* < .05 and ^∗∗^*P* < .01 between the groups. ^∗^shSTAT3 group compared with that of the shNC group. (b) The cell proliferation activity was measured with a CCK8 assay after OGD/R and treated with Fe or Fer-1. ^∗^*P* < .05, ^∗∗^*P* < .01, and ^∗∗∗^*P* < .001 between the groups. ^∗^shSTAT3 group compared with that of the shNC group. (c, d) The level of GSH and MDA in STAT3 knockdown cells after the OGD/R model; ^∗^*P* < .05, ^∗∗^*P* < .01, ^#^*P* < .05, and ^##^*P* < .01 between the groups. ^∗^OGD/R group compared with the control group; ^#^shSTAT3 group compared with that of the shNC group. (e) Relative level of GPX4 protein expression after interfering with STAT3. ^∗^*P* < .05, ^∗∗^*P* < .01, ^∗∗∗^*P* < .001, ^#^*P* < .05, ^##^*P* < .01, and ^###^*P* < .001 between the groups. ^∗^OGD/R group compared with the control group; ^#^shSTAT3 group compared with that of the shNC group.

To further elucidate whether STAT3 activation contributes to ferroptosis resistance, we used the shRNA lentivirus to knockdown STAT3 expression in MLE12 cells. The results showed that STAT3 interference significantly reduced the level of STAT3 and downregulated SLC7A11 expression although pSTAT3/STAT3 did not change significantly (Figure 7(a)). As a result, we found that cell death was more severe than that observed in MLE12 cells transfected with the NC lentivirus (Figure 7(b)). Moreover, STAT3 interference significantly increased the reduced level of GPX4 (Figure 7(e)) and GSH (Figure 7(c)). The level of MDA (Figure 7(d)) increased to a greater extent following OGD/R.

### 3.8. STAT3 Overexpression Upregulates SLC7A11 and Ameliorates OGD/R-Induced MLE12 Ferroptosis

**Figure 8:**
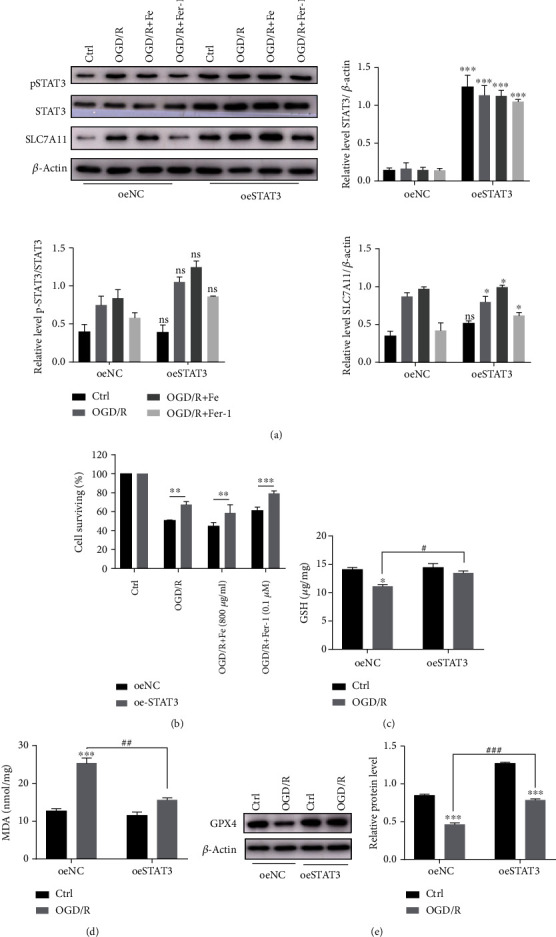
STAT3 overexpression may alleviate the OGD/R-induced lung epithelial cell ferroptosis. Cells transfected with the STAT3 overexpressing lentivirus were subjected to OGD/R. (a) Relative level of STAT3, pSTAT3, and SLC7A11 protein expression after STAT3 overexpression. The pSTAT3 intensity was normalized to the corresponding level of STAT3. The STAT3 and SLC7A11 intensity were normalized to the corresponding level of *β*-actin, and the changes are shown in the histograms. ^∗^*P* < .05, ^∗∗^*P* < .01, and ^∗∗∗^*P* < .001 between the groups. ^∗^oeSTAT3 group compared with that of the oeNC group. (b) The cell proliferation activity was measured using a CCK8 assay after establishing OGD/R and treating with Fe or Fer-1. ^∗^*P* < .05, ^∗∗^*P* < .01, and ^∗∗∗^*P* < .001 between the groups. ^∗^oeSTAT3 group compared with that of the oeNC group. (c, d) The level of GSH and MDA in the STAT3 overexpressing cells after OGD/R; ^∗^*P* < .05, ^∗∗^*P* < .01, ^∗∗∗^*P* < .001, ^#^*P* < .05, ^##^*P* < .01, and ^###^*P* < .001 between the groups. ^∗^OGD/R group compared with the control group; ^#^oeSTAT3 group compared with that of the oeNC group. (e) Relative level of GPX4 protein after overexpressing STAT3. ^∗^*P* < .05, ^∗∗^*P* < .01, ^∗∗∗^*P* < .001, ^#^*P* < .05, ^##^*P* < .01, and ^###^*P* < .001 between the groups. ^∗^OGD/R group compared with control group; ^#^oeSTAT3 group compared with that of the oeNC group.

Moreover, we overexpressed STAT3 using a STAT3-overexpressing lentivirus. Although the level of SLC7A11 and pSTAT3 did not increase under normal conditions and the percent of pSTAT3/STAT3 did not change in the different groups; their expression levels of protein increased after OGD/R model (Figure 8(a)). With increased phosphorylated STAT3 and SLC7A11, the cell damage caused by OGD/R was significantly decreased (Figure 8(b)). Furthermore, STAT3 overexpression alleviated the decrease in the level of GPX4 (Figure 8(e)), GSH (Figure 8(c)), and MDA (Figure 8(d)) after OGD/R. Together, these results indicate that STAT3 activation regulates SLC7A11 to inhibit ferroptosis and plays a protective role in OGD/R-induced ferroptosis.

## 4. Discussion

Since ferroptosis was identified as a new form of RCD [12, 35, 36], it has been closely correlated with human diseases. Four key factors (i.e., iron, PUFAs, oxygen, and a reduction in antioxidants) are indispensable for ferroptosis induction [37]. Previous studies have shown that ferroptosis is involved in IR injury in the liver [38], brain [39], kidney [9], and heart [10]. Thus, we hypothesized that ischemia/reperfusion or hypoxia/reoxygenation may be an important process that facilitates the occurrence of ferroptosis.

In this study, we systematically studied the role of ferroptosis in ALI induced by IIR. In IIR-ALI model, the endogenous level of ferric, ferrous iron, and MDA content in lung tissue increased significantly, which indicated the occurrence of ferroptosis in pulmonary epithelial cells considering the typical changes of mitochondria simultaneously. Treating with ferrostatin-1, a specific ferroptosis inhibitor, was found to rescue IIR-ALI by alleviating the histological injury to the lung, improving pulmonary epithelial cell viability, and restoring epithelial barrier function. Furthermore, the expression of GPX4, a key ferroptosis factor, was also recovered by ferrostatin-1, supporting the presence of ferroptosis in IIR-ALI and OGD/R-induced MLE12 cell damage. These findings are consistent with the results reported by Li et al. [4].

Nrf2 is a crucial redox-sensitive transcription factor that plays a key role in cellular cytoprotection against oxidative and electrophilic stress [17]. Nrf2 status is reported as a key element in determining the therapeutic responses to ferroptosis-targeted therapies in hepatic tumors [25]. However, its intrinsic mechanism in ferroptosis-mediated ALI has not yet been clarified. In our previous study, we found that Nrf2 was increased in IIR-ALI. As a key regulator of antioxidant stress, it could inhibit ferroptosis by upregulating the expression of SLC7A11 and HO-1, thus playing a protective role in IIR-ALI [24]. Surprisingly, STAT3 was intensively activated in the *Nrf2* silencing mice than that of WT mice in the IIR model, implying that Nrf2 might potentially make influence on STAT3 signaling.

Functioned as an oxidative responsive transcriptional factor, STAT3 was reported to play an active role in stress-related ferroptosis [40]. Liu et al. reported that treatment with a STAT3 inhibitor reactivated ferroptosis in the cells and consequently increased the sensitivity to cisplatin [41]. Additionally, Brown et al. showed that *α*6*β*4-mediated activation of Src and STAT3 suppressed the expression of ACSL4, an enzyme required for ferroptosis [42]. In our study, pharmacologic activation of STAT3 was found to alleviate MLE12 cell damage in the OGD/R model, while pharmacologic inhibition of STAT3 activation could aggravate MLE12 cell damage induced by OGD/R, which demonstrated the protective role of STAT3 in ferroptosis induced by OGD/R. On the other hand, overexpression of STAT3 promoted the expression of SLC7A11, while downregulation of STAT3 decreased the expression of SLC7A11, demonstrating the potential regulation effect of STAT3 on SLC7A11.

The *SLC7A11* gene belongs to the solute transport family and encodes a cystine/glutamate xCT transporter, which is a key gene involved in regulating “iron overload-ferroptosis” [12]. Qian et al. found that the downregulation of xCT exacerbated lung injury due to its key role in regulating cysteine uptake, leading to the maintenance of intracellular cysteine and GSH pools; thus, inhibiting xCT expression impairs GSH synthesis, resulting in severe oxidative stress [43]. Our findings are consistent with this previous research. We found that either ferroptosis inhibition or STAT3 overexpression significantly alleviates IIR-ALI, which suggests a potential therapeutic approach for the treatment and/or prevention of ischemic lung damage.

**Figure 9:**
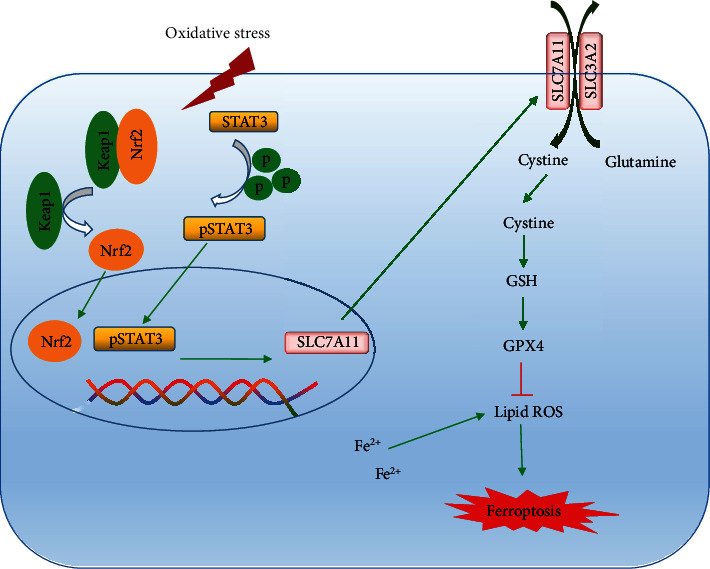
The roles of Nrf2 and STAT3 in regulating ferroptosis in IIR-ALI.

Both *Nrf2* and *STAT3* are antioxidant response elements; when cells are under oxidative stress, they promote the expression of downstream target genes (e.g., SLC7A11) to enhance cell resistance. Some findings have suggested that the transcriptional activity of Nrf2 and STAT3 may co-regulate the expression of ferroptosis-related genes such as xCT [40]. Our results indicate that Nrf2 and STAT3 might be co-responding to ferroptosis and collaborate to regulate SLC7A11 in ferroptosis. As two crucial antioxidant regulators, STAT3 could further be activated when knocking out Nrf2, so as to exert the protective role to resist excessive oxidative damage in IR-induced ALI (graphic summary, Figure 9).

## 5. Conclusions

Taken together, we confirm the involvement of ferroptosis in intestinal IR injury and reveal that ferrostatin-1 protects against IR injury and restores the lung epithelial barrier function by inhibiting ferroptosis. Nrf2 and STAT3 co-regulate SLC7A11 to alleviate ferroptosis in IIR- ALI. Future investigations will focus on the crosstalk of Nrf2 and STAT3 on ferroptosis and extend inhibition strategies to IIR-ALI treatment.

## Data Availability

The data used to support the findings of this study are included within the article.
